# Carbapenem resistant *Klebsiella pneumoniae* isolates at a tertiary hospital in Cape Town, South Africa, are dominated by specific local clones rather than previously described international lineages

**DOI:** 10.1371/journal.ppat.1013859

**Published:** 2026-01-16

**Authors:** Kedišaletše Moloto, Mae Newton-Foot, Angela Dramowski, Stefany Ayala-Montaño, Ifeoluwa Akintayo, Andrew Whitelaw, Sandra Reuter

**Affiliations:** 1 Division of Medical Microbiology, Department of Pathology, Faculty of Medicine and Health Sciences, Stellenbosch University, Stellenbosch, South Africa; 2 National Health Laboratory Service, Tygerberg Hospital, Cape Town, South Africa; 3 Department of Paediatrics and Child Health, Faculty of Medicine and Health Sciences, Stellenbosch University, Cape Town, South Africa; 4 Institute for Infection Prevention and Control, Medical Center University of Freiburg, Freiburg, Germany; Catalan Institute for Water Research (ICRA), SPAIN

## Abstract

Carbapenem-resistant *K. pneumoniae* (CRKP) are increasingly prevalent pathogens in hospital settings worldwide. In early 2019, an outbreak of carbapenem-resistant Enterobacterales (CRE) at a tertiary hospital in South Africa triggered a retrospective investigation to determine local CRKP molecular epidemiology. The evolution of CRE at the institution progressed from sporadic cases in 2016–2017 to establishment of CRKP as an endemic pathogen by 2020. Of 778 clinical and carriage CRKP isolates (2016–2020), 413 (53%) were collected and sequenced. Sequence type (ST) 2621 (164, 40%) and ST39 (164, 40%) predominated with several minor STs making up the remainder. The majority of ST2621 associated with *bla*_OXA-181_ were isolated from adult patients (59%) and clinical samples (84%). ST39 with *bla*_NDM-1_ was predominantly identified in carriage isolates (76%) from neonatal (57%) and paediatric (38%) patients. The establishment of these unique CRKP clones distinct from the globally dominant epidemic lineages suggests local acquisition of carbapenemase genes with subsequent institutional propagation. Strengthening of infection prevention practices and antibiotic stewardship programmes at this institution is critical to reduce CRKP transmission and curtail evolution of future antimicrobial resistant clones.

## Author summary

Carbapenem resistant *K. pneumoniae* (CRKP) have emerged as a cause of hospital and community acquired infections, posing a significant threat to public health, and have hence been listed as a priority pathogen by the World Health Organization (WHO). In this study we investigated the molecular epidemiology of CRKP and found that local endemic CRKP was dominated by two sequence types which had both been present since 2016. These may be local clones that have acquired carbapenemase genes and become disseminated in our setting, a marked difference from the intercontinental spread observed for other successful CRKP lineages. Our findings will assist in improved monitoring and identification of local CRKP, which will aid in preventing nosocomial infections in vulnerable patients by contributing to guidelines for infection prevention and control practises. Furthermore, this implies that in other understudied locations, CRKP also need to be investigated, to determine how local clones may differ and how they arise. This can in turn teach us about patterns of evolution of successful clones, especially with respect to particular plasmid types or carbapenemase genes.

## Introduction

*Klebsiella pneumoniae* is a Gram-negative bacillus that causes healthcare-associated infections (HAI) such as bloodstream, pneumonia and urinary tract infections in immunocompromised or often hospitalized individuals [1,2]. In recent decades, Gram-negative bacteria producing extended-spectrum beta-lactamases (ESBLs) have become established as major pathogens, in both HAI and community-acquired infections [3]. These resistance genes confer the ability to hydrolyse cephalosporins, monobactams and penicillins, limiting effective treatment options for ESBL-producing bacterial infections to the carbapenem class of antibiotics [4]. Subsequently, the overuse of these ‘antibiotics of last resort’ has selected for carbapenem resistance (CR), with production of carbapenemases as the major resistance mechanism.

Carbapenemase enzymes were first identified in Enterobacterales in the 1980s and have since disseminated globally [5]. Among Enterobacterales, clinically significant carbapenemases include the Ambler molecular class A (*bla*_KPC_), class B metallo-beta-lactamases (*bla*_NDM_, *bla*_IMP_, *bla*_VIM_), and class D oxacillinases (*bla*_OXA-48_) types. These carbapenemases have all been reported in *K. pneumoniae* isolates and are also frequently associated with serious HAI and outbreaks. The emergence of carbapenem-resistant *K. pneumoniae* (CRKP) strains has become a challenge for public health globally [6].

The major burden of antimicrobial resistance (AMR) is due to the global spread of certain clones referred to as high-risk clones [7]. One of the most established global high-risk MDR clones of *K. pneumoniae* is the clonal lineage sequence type (ST) 258/512 consisting mainly of ST258 and ST512 [8,9]. This dominant lineage has been reported in European and Western countries such as Greece, Norway, Sweden, Italy and Canada and the USA [8,9]. Certain emerging high-risk clones such as ST307 and ST147 have been reported as vehicles for the spread of AMR determinants and need further monitoring [7]. Since the epidemic dominant lineage ST258/512 is associated with the presence of blaKPC-2 and *bla*_KPC-3_, *K. pneumoniae* carbapenemase (KPC) is the most common carbapenemase. However, in the African context *bla*_NDM_ and *bla*_OXA-48_ are the most common and associated with ST101, with limited reports of ST258 and ST307 [10,11]. ST16 and ST147 have been reported in CRKP isolates co-harbouring *bla*_NDM_ and *bla*_OXA-48-like_ genes in Thailand [12]. A study in China reported that the most prevalent strains were ST11 and ST15, which are known international high-risk CRKP clones reported mainly from Asia and Europe, and responsible for nosocomial transmission and various care centre outbreaks [13–15]. A comparison of CRKP sequences from South America found that ST14 followed by ST147, ST11 and ST348 were the most common STs among Peruvian isolates, whereas ST11 and ST258 were dominant in isolates from Argentina, Brazil and Colombia [16].

The first South African laboratory confirmed case of carbapenem resistance was reported in 2011 from an academic hospital in Johannesburg, in a patient with CR *Enterobacter cloacae* [17]. In May 2012, a tertiary academic hospital in Cape Town reported one of the first laboratory-confirmed outbreaks of *K. pneumoniae* expressing OXA-181, belonging to ST14 and linked to two patients admitted to the haematology ICU [18]. Until 2015, blaNDM was the predominant carbapenemase reported in South Africa [19], but more recent epidemiological reports (2015–2018) have noted a shift to predominance of *bla*_OXA-48_ and its variants [20].

In late 2018, an outbreak of CRE in the neonatal unit at Tygerberg Hospital (TBH) [21], a tertiary hospital in Cape Town, South Africa, triggered a retrospective epidemiological investigation. Although the neonatal unit had been the first clinical area at the institution to declare a CRE outbreak, sporadic CRE cases had been identified in the institution’s adult and paediatric wards since 2016. We aimed to investigate and describe the molecular epidemiology of CRKP at the institution, progressing from sporadic cases in 2016–2017 to establishment of CRKP as an endemic pathogen at the institution by 2020.

## Results

### Demographic and isolate data

**Fig 1:**
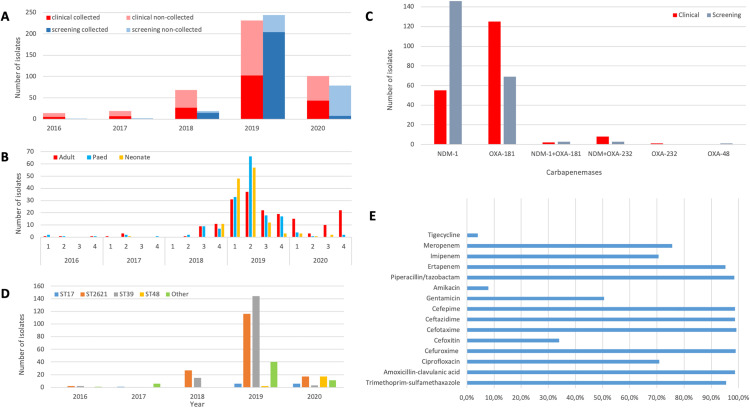
Overview of the CRKP collection from TBH. **A:** Carbapenem resistant *K. pneumoniae* isolates collected and sequenced (solid bars) vs the total population of CRKP (“non-collected”, lighter bars) from 2016 to 2020. **B:** Distribution of isolates collected from patients in adult, paediatric and neonatal wards from 2016 to 2020. **C**: Carbapenemase genes detected among the CRKP isolates. **D**: Prevalent carbapenem resistant *K. pneumoniae* sequence types from 2016 to 2020. **E:** Antibiotic susceptibility profile of *K. pneumoniae*.

Clinical and laboratory data received from the Western Cape’s Provincial Health Data Centre showed that a total of 778 CRKP isolates (759 patients) were recorded at the institution between 2016 and 2020. However, isolates could only be obtained from 413 CRE episodes (399 patients) across the adult (152, 36.8%), paediatric (141, 34.1%), and neonatal (120, 29.1%) wards. Fourteen patients had duplicate isolates of which the majority (12/14) had different strains in the two samples collected. The ward distribution and sex of CRKP infected/colonized patients and specimen type distribution of the collected isolates were nonetheless representative of the total population (Table 1; S1 Table). The majority of CRKP isolates (307, 74.3%) were collected in 2019, when the outbreak was declared (Fig 1A), with similar proportions of clinical and carriage isolates (Table 1). 76.6% (200/261) isolates from paediatric and neonatal patients were isolated from carriage samples collected during the outbreak period in early 2019 (Fig 1B). Fewer isolates, in particular isolates from screening samples, were detected and recovered in 2020 due to the SARS-Cov-2 pandemic, where clinical services were reprioritised, and research activities curtailed due to lockdowns.

**Table 1:** Clinical metadata of the total isolates (based on data from Provincial Data Centre) and the isolates stored and analysed during the study period.

	Total isolates (n = 778) (%)	Stored isolates (n = 413) (%)
**Gender**		
**Male**	391 (50.3)	214 (51.8)
**Population group**		
**Adult**	339 (43.6)	152 (36.8)
**Paediatric**	235 (30.2)	141 (34.1)
**Neonate**	196 (25.2)	120 (29.1)
**Not stated**	8 (1.0)	–
**Source**		
**Clinical**	357 (45.9)	186 (45.0)
**Carriage**	421 (54.1)	227 (55.0)
**Specimen type: Clinical**		
**Urine**	106 (13.6)	42 (10.2)
**Blood culture**	80 (10.3)	79 (19.1)
**Respiratory**	55 (7.1)	28 (6.8)
**Tissue**	36 (4.6)	15 (3.6)
**Swab**	39 (5.1)	11 (2.9)
**Catheter**	20 (2.6)	6 (1.5)
Other*	22 (2.8)	6 (1.5)
**Specimen type: Carriage**		
**Rectal swab**	345 (44.3)	178 (43.1)
**Stool**	74 (9.5)	47 (11.4)

* Other: aspirate (stored: 2), Fluid (total: 15, stored: 2), Ventriculoperitoneal shunt (total: 3). CSF: cerebrospinal fluid (total: 4; stored: 2)

### Detection of antibiotic resistance genes

Carbapenemase genes *bla*_NDM_ and *bla*_OXA-48-like_ were detected in 375 (91.0%) isolates by real-time PCR and in 402 isolates (97.3%) by whole genome sequencing (WGS). Of the 27 isolates where a carbapenemase was not detected by PCR, 12 harboured *bla*_NDM-1_ and 15 harboured *bla*_OXA-181._ The predominant genes were *bla*_OXA-181_ (194, 46.9%) and *bla*_NDM-1_ (191, 46.2%). A combination of *bla*_NDM-1_ and *bla*_OXA-181_ was seen in four isolates (1.0%), *bla*_NDM-1_ and *bla*_OXA-232_ in 11 isolates (2.7%), and *bla*_OXA-232_ and *bla*_OXA-48_ in one isolate each (0.2%) (Fig 1C). Interestingly, most isolates with *bla*_NDM-1_ were from carriage samples (146, 72.6%), whereas those with *bla*_OXA-181_ were mostly clinical isolates (125, 64.4%) (Fig 1C). More carbapenemase genes were detected with WGS than with real-time PCR, with WGS showing a higher degree of discrimination determining the gene variant, i.e. *bla*_OXA-181_ which is a variant of *bla*_OXA-48_ with four amino acid substitutions but with the same hydrolytic properties [22]. The most detected resistance genes (other than carbapenemases) were *bla*_CTX-M-15_ (95.4%), *strA* (80.0%) and *strB* (79.0%) conferring resistance to extended-spectrum beta-lactam antibiotics and aminoglycosides, respectively.

Antimicrobial susceptibility testing (AST) data was available for 394 isolates (96.4%). The isolates showed high rates of resistance (>95%) to amoxicillin-clavulanic acid (389, 99.5%) piperacillin-tazobactam (388, 98.5%), cefotaxime/ceftriaxone (391, 99.2%), ceftazidime (389, 98.7%), cefepime (389, 98.7%) and trimethoprim-sulfamethoxazole (376, 95.4%) (Fig 1E). They were least resistant to amikacin (31, 7.6%) and tigecycline (16, 4.1%). Only 154 isolates were tested for colistin resistance, with 142 (92.2%) susceptible, 10 (6.5%) intermediate and 2 (1.3%) resistant based on CLSI criteria in use at the time isolates were obtained. Non-susceptibility (resistant and intermediate) to carbapenems was 99.7% (336/337) ertapenem, 91.0% (315/346) meropenem and 83.7% (288/344) imipenem (S1 Table/S1E Fig).

### Strain typing

Although MLST showed a diverse population consisting of 25 sequence types, ST2621 and ST39 were overwhelmingly dominant in the collection (S1 Table), represented by 164 isolates (39.6%) each. These were followed by ST48 (19, 4.6%), ST17 (13, 3.1%), ST2497 (9, 2.2%), ST502 (7, 1.7%) and ST334 (6, 1.4%) (Fig 1D). All isolates had a virulence score of either 1 (89.3%, positive for yersiniabactin (ybt) only) or 0 (10.7%, negative for ybt, colibactin (clb), and aerobactin (iuc)).

**Fig 2:**
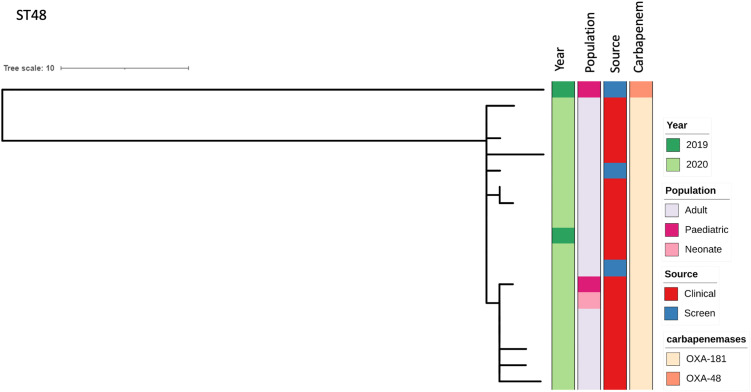
Phylogenetic tree for ST48. Year of isolation, source (clinical or carriage sample), population (adult, neonate, paediatric), and the carbapenemase genes detected are shown.

Both ST2621 and ST39 were first detected in 2016 (2 isolates each), and then predominated in 2018 and 2019 (ST2621: 27 (64.3%) and 115 (38.2%) respectively; ST39: 15 (35.7%) and 140 (46.5%) respectively). ST48 was first detected in 2019 (2, 0.7%), and in 2020 (17, 32.7%), it was more common than ST39 (3, 5.8%) and as common as ST2621 (19, 36.5%), which had decreased (Fig 1D). All ST48 isolates were *bla*_OXA-181_ producers, except for the first ST48 isolate from 2019, which produced *bla*_OXA-48_ (Fig 2). This ST was predominantly in adult patients (16, 84.2%) and from clinical samples (15, 79.0%). The isolates were closely related, separated into one clade consisting of all OXA-181 producers (0–7 SNPs), with the single OXA-48 producer differing from the other isolates by >80 SNPs (Fig 2). The isolates did not seem to be epidemiologically linked, with the first two isolates in 2019 being from a paediatric ward in March and later in an adult ward in December. The following isolates were more epidemiologically linked, only seen from August 2020, with only one isolate from the neonatal unit and the rest from adult wards (S1 Table).

Interestingly, ST334 was only seen in 2016 (one isolate) and almost a year later in 2017 (five isolates) with minor variations of 0–5 SNPs among all isolates, and not again in the subsequent years. The 2017 isolates were seen in March in an adult ward then later in May (four isolates) in paediatric and adult wards. All ST334 isolates were *bla*_NDM-1_ producers (S1 Fig). Surprisingly, of the previously described dominant global epidemic lineages, we only found a single ST307 isolate, which also did not carry any carbapenemase gene but was resistant to ertapenem, imipenem and meropenem. We focused our further analysis on the two dominant clones to understand them better.

### Characteristics of ST39

**Fig 3:**
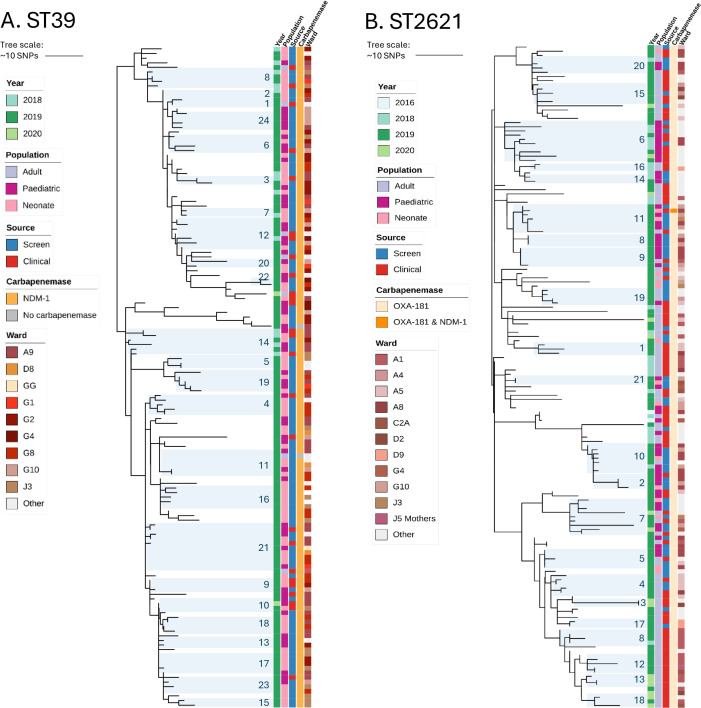
Phylogenetic tree of the endemic expansion of ST39 (A) and ST2621 (B). Year of isolation, source (clinical or carriage sample), population (adult, neonate, paediatric), the carbapenemase genes detected and the wards are shown. Clusters based on a 2-SNP cutoff are shown in shaded ranges.

**Fig 4:**
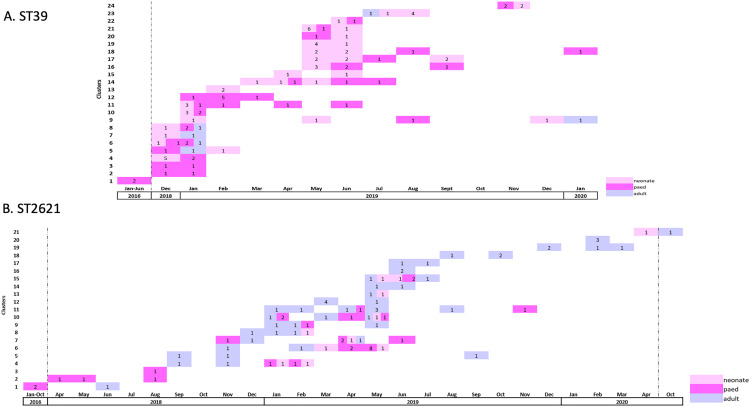
Transmission chains of ST39 isolates (A) and ST2621(B). Dotted lines indicate months/years where no clustered isolates were collected.

ST39 was clustered into three distinct clades (S2 Fig; https://microreact.org/project/5ZJRSb6bshjY5f5fmMhzY8-cresast39). The three oldest ST39 isolates (two from 2016, one from 2018) formed a basal clade to the rest of the isolates, and a second clade of 13 isolates with little variation was observed (S2 Fig, inset), with two outlying sequences. The rest of the isolates formed a clonal expansion with minor variations in SNP distances, with the first isolate in June 2019 (S2 Fig “endemic expansion”, S3A Fig). The majority of the ST39 isolates were obtained from neonatal (92, 56.1%) and paediatric (64, 39.0%) patients and mostly in carriage samples (128, 78.0%). However, no pattern is evident with respect to clustering by time or patient type, as isolates are intermingling (Fig 3A). Given the diversity between the isolates and the overall structure of the phylogeny, this clone may have been locally circulating with ongoing evolution outside of the sampled population within the institution and/ or surrounding communities. Using a two SNP threshold between isolates from different patients to identify transmission chains, we identified 24 ST39 clusters (Fig 4A), ranging from two to eight isolates. Eighteen (75.0%) of the clusters contained only isolates from neonatal and paediatric patients. The first cluster was from 2016 with two clinical isolates (one in January, one in June) from paediatric patients. The longest lasting cluster was one year (January 2019 to January 2020, cluster 9) with five isolates. This cluster included isolates from three neonates and a paediatric patient from 2019 and one adult patient in 2020.

ST39 was associated with O locus OL2α.1, with O locus type O1αβ,2β in all isolates. The endemic expansion of ST39 (n = 147, 89.6%) had the K locus KL2 (wzi2) and a virulence score of 1, with the yersiniabactin *ybt*16 carried on ICE*Kp*12. Of the other remaining isolates, three had the locus KL149 (wzi452) and a virulence score of 1 with *ybt*14 on ICE*Kp*5, thirteen had the K locus KL62 (wzi62) and one isolate had KL23 (wzi83), the latter both with a virulence score of 0 (S1 Table Virulence summary).

### ST39 in a global context

**Fig 5:**
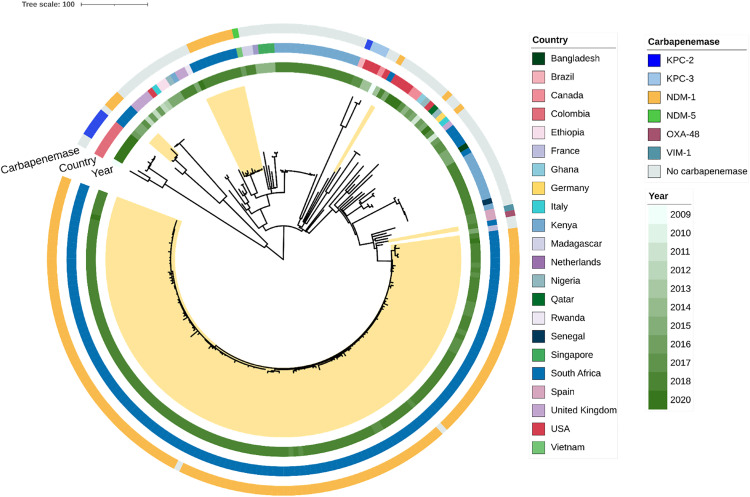
Phylogeny of a global selection of ST39 isolates. CRKP of this study are highlighted in yellow. Year and country of isolation, as well carbapenemase genes are shown. The ST39 isolates from the institution outside the expansion fall into distinct clones, with no close links to any global isolates.

To put this CRKP collection and population into context, we downloaded the sequences of an additional 85 isolates representing the diversity within this ST on a global scale through inclusion of isolates from Pathogenwatch (Fig 5; interactive version (https://microreact.org/project/k5xuuDEJurmbW4tbUuMzqw-cresaglobalst39final230523). African isolates are dispersed around the tree, however the isolates from Cape Town are distinct from the background population. Some geographic clustering is evident, and except for the TBH isolates, *bla*_KPC_ seems to be a more common carbapenemase. However, both these observations might be artefacts from subsampling. The isolate closest to the local expansion is from France, but at a considerable genetic distance rather than a direct precursor; this isolate is also carbapenem susceptible. This global view supports the notion that this clone evolved locally. Furthermore, the plasmid content of this clone (S3 Fig), showed that CRKP isolates from the institution, irrespective of the three observed clades, carried IncFII and IncFIB replicons, which were almost absent in the subsampled global collection. The expansion clade also had a ColRNAI replicon, which is more common and present in carbapenemase negative samples from the global collection. Sequence analysis indicated that the *bla*_NDM-1_ gene is in a cassette flanked by transposable elements, as it assembled into a contig of its own with no means of placing it in a plasmid backbone based on short-read data alone. An exception is a clinical isolate from 2019 in the clonal expansion, in which the *bla*_NDM-1_ gene is located on the IncFIB plasmid. Better resolution with long read sequencing would be needed to gain a better understanding of the genetic content flanking this particular resistance gene. The presence of IncFIB plasmids in all TBH isolates, however, strongly suggests that this is the plasmid carrying the *bla*_NDM-1_ gene. Since this plasmid type is rare and only found in a single isolate outside of Cape Town in our subsampled global ST39 collection, we cannot speculate as to the origin of this plasmid, its transfer into and the evolution of this particular clone.

### Characteristics of ST2621

In contrast to ST39, ST2621 has not been observed in fully sequenced CRE collections thus far. The ST has been submitted to the MLST database in 2017, however neither Pathogenwatch, an extensive repository of over 48,000 *K. pneumoniae* isolates (https://pathogen.watch/; latest genome from September 2023), nor the genome collection of National Center for Biotechnology Information (NCBI) (over 54.000 *K. pneumoniae*) show an isolate match for this particular ST. Further analysis of the MLST loci revealed that it is a single locus variant of ST17, which in contrast has been widely reported and described [23,24]. We therefore reconstructed a phylogeny of the local ST17 complex (S4 Fig;https://microreact.org/project/edU1Gx4U1mpafiwYak8Ha9-copy-of-cresaonlyst172621230711), and found that the ST17 isolates formed two separate clades, and that ST2621 formed a single expansion with multiple clades (Fig 3B). The two oldest sporadic cases of ST17 complex are of ST2621, indicating a possible circulation of this clone prior to the declaration of the outbreak of the successful CR lineage in 2019. *bla*_OXA-181_ was the predominant carbapenemase gene associated with this ST, being detected in all isolates, with one isolate having an additional *bla*_NDM–1_ gene.

The majority of the ST2621 isolates were from adult patients (95, 58%) and from clinical samples (98, 60%). There is no strong pattern evident with respect to clades clustering by patient type, or time, but rather the clone is spread throughout the institution (Fig 3B). Similar to ST39, the diversity within the ST2621 expansion within TBH might reflect a locally circulating clone with evolution outside of the sampled population being repeatedly introduced into the institution rather than a single, continuous transmission chain. After defining transmission clusters using a 0–2 SNP difference, twenty-one ST2621 clusters were identified containing two to fourteen isolates each (Fig 4B). Eight clusters (38.1%) were from adult wards only, and eleven (52.4%) were a combination of adult and children’s wards (neonatal and paediatric). The remaining two were only from the children’s wards. The longest lasting cluster exceeded two years; this cluster consisted of three clinical isolates, two of which were isolated from paediatric patients in 2016 (one January, one in October) and one from an adult patient isolated in June 2018. All ST17 (n = 13) and ST2621 had the K locus KL25 (wzi141) and O locus OL5, and a virulence score of 1 with the yersiniabactin *ybt*14 carried on ICE*Kp*5. (S1 Table_Virulence summary).

### ST2621 in a global context

**Fig 6:**
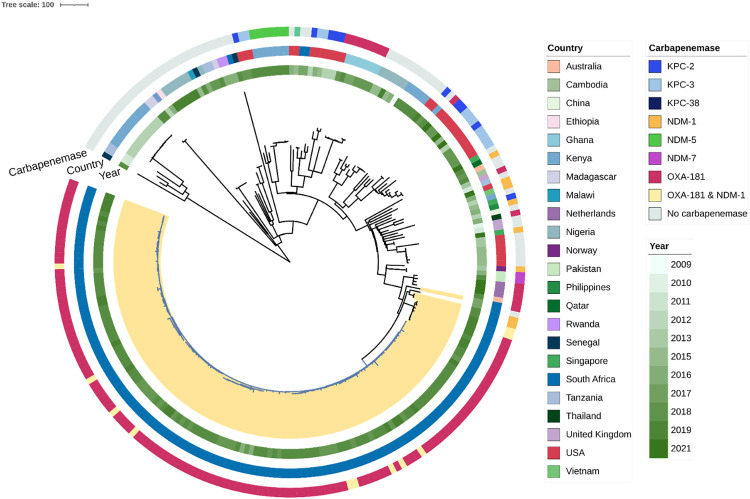
Phylogeny of a global selection of ST17 Complex isolates. CRKP of this study are highlighted in yellow. Year and country of isolation, as well carbapenemase genes are shown. The branches of the local expansion are blue.

We then contextualized our isolates within a larger ST17 collection (Fig 6; interactive version https://microreact.org/project/gw3jhssF7We1ybi3ppixKy-globalcresast172621230906). From Pathogenwatch, we selected the main branch on which ST2621 as a single locus variant of ST17 is located. This branch also contains globally distributed isolates, and we focussed on human isolates and included samples from Africa as continental representatives for contextualisation. In branches containing clonal isolates and therefore both suspected outbreak samples as well as redundancy, we selected one to two isolates. Isolates from Africa are found on all clades of the tree. Interestingly, the isolate closest to the ST2621 from Cape Town originates from a local study into the mortality associated with third-generation cephalosporin resistance in bloodstream infections at the same institution [25]. This isolate was a true ST17, isolated in 2017, carbapenem susceptible and therefore is not the direct founder of the ST2621 clonal expansion. It does, however, lend further strong support to a locally evolved clone. This complex showed a larger variety of commonly found clones than those in ST39 (Fig 6). Isolates from the institution most consistently carried IncFIB, ColKP3, and IncX3 plasmids, though other plasmid types were also common (IncR, IncFII, IncFIA, and ColRNAI). We observed that ColKP3-IncX3 plasmids have a strong correlation with the carbapenem-resistance profile (*bla*_OXA-181_) even at a global level outside the TBH isolates. Short read sequencing indicated that the *bla*_OXA-181_ gene is carried on the ColKP3 plasmid, as the replicon type and gene were always found on the same contig (S5 Fig). A hybrid plasmid of ColKP3-IncX3 with *bla*_OXA-181_ has been characterized in *K. pneumoniae* and *E. coli*, indicating that wide dissemination is possible even though this plasmid is scarce in a global context thus far [8,26]. Further long read sequencing would be needed to characterize the plasmids in our collection as well as those of the global collection in more detail, to determine the exact structure and potential sharing that may have given rise to the successful local lineage.

## Discussion

In this study, we describe the molecular epidemiology of CRKP at a large tertiary hospital in Cape Town, South Africa, including both infection and carriage isolates. Approximately half (53.1%) of all the isolates cultured (2016–2020) were stored for analysis. Overall, the stored and subsequently sequenced isolates provide an adequate representation of the total study population during that time period, however, they may not have accurately represented the genetic diversity. The longitudinal design of the study permitted evaluation of trends in CRE evolution at the institution over an extended period.

In 2016 and 2017, the majority of CRKP isolated were identified from clinical samples (Fig 1A). There was a marked increase in isolation of CRKP from both clinical and carriage samples from late 2018 onwards, leading to the declaration of a CRKP outbreak in the institution’s neonatal unit. The increased CRKP detection rate was driven by an increased number of CRKP carriage isolates from the paediatric and neonatal wards, as well as increased numbers of CRKP clinical isolates from adult patients. This suggests that the increase in CRKP in the adult wards may have been underappreciated at the time, possibly owing to a large population of adult inpatients distributed across a range of wards and clinical disciplines. The differences in screening practices between adult and neonatal wards was due to a combination of financial constraints (smaller paediatric platform thus screening less costly overall), infrastructural concerns (limited capacity to carry out preventive measures such as cohorting and isolation of patients on adult platform), and differences in perceived benefit of screening across clinical services.

The analysis of the CRKP population at the institution showed that the CRKP were diverse and comprised 24 individual sequence types, 20 of which were represented by <5 isolates. None of these were the commonly seen international clones. The collection was dominated by two sequence types, ST39 and ST2621, with 40.0% of the isolates belonging to each of these STs. The CRKP mainly harboured *bla*_NDM-1_ and *bla*_OXA-181_, which are commonly detected carbapenemase genes in South Africa [10,27–29]. The origin of these local clones is unknown, however given the genetic diversity present within the institution and the population structure, we suspect that they may have been circulating outside of our clinical setting for some time before spilling over into the institution. We do not have any data predating 2016 from private hospitals or other public hospitals and therefore do not know which strains are circulating there and how ST2621 and ST39 could have been introduced into the institution. A previous study into ESBL-producing Enterobacterales including *K. pneumoniae* [25] found three ST17 and seven ST39 at the institution, but the global analysis including these isolates only highlighted a single ST17 isolate as closest to the current dominant lineage ST2621.

Using a threshold of two SNPs to define transmission, we revealed the presence of multiple transmission clusters within each ST. However, this analysis likely underestimates the amount of in-house transmission, as approximately 50% of isolates could not be recovered. Inclusion of these missing isolates may result in the merging of some or all of the currently identified SNP clusters. This is already evident by some of the clusters being close to other clusters on the tree but exceeding the defined threshold. The two SNP threshold for identifying transmission events is very stringent and was chosen to reliably identify transmission events in the absence of any supporting data on patient movements. Using a threshold of 10–25 SNPs would likely have also resulted in merging of some clusters. Additionally, there may be patients with CRE from whom no samples were collected, and there may also be unidentified environmental reservoirs contributing to the spread. It is plausible that ST2621 and ST39 were introduced into the hospital around 2016 or slightly earlier and have since undergone transmission and evolution, rather than being reintroduced multiple times into the hospital.

The global ST39 analysis showed the clear distinction of global as well as other African isolates from the expanding lineage at the institution. The local lineage also stands out with its carriage of *bla*_NDM-1_ and plasmid repertoire. In Europe, ST39 has recently been a source of concern in KPC-2 CRKP strains resistant to ceftazidime-avibactam [30]. Another study reported ST39 CRKP with KPC-2 and VIM-1 in Greece [31]. This contrasts with the ST39 strains in this study, which were found to be NDM-1 producers. This is suggestive of potential plasmid or transposon transfer contributing to the rise of successful lineages. The global spread of NDM remains one of the most worrisome antimicrobial resistance events caused by metallo-β-lactamases. *bla*_NDM_ genes are dominant in *K. pneumoniae* in certain global lineages such as ST11, ST14, ST15, ST101 and ST147 [32]. The transmission of ST39 within the institution was restricted in time, with the majority of the isolates being identified from December 2018 and with a large increase in detection rates from January 2019. This was due to the implementation of screening practices, initially on the TBH neonatal wards, then later in the TBH paediatric wards. Several contingency measures were then put into place to contain the outbreak in these wards. These included intensified infection control and cleaning of the hospital environment, clinical equipment and incubators, opening of additional areas for more neonatal beds, and assigning additional staff to care for babies who required isolation. This response appears to have limited further spread of this ST leading to lower numbers of ST39 in late 2019 and 2020.

In contrast, ST2621 is a rare ST. It is a single locus variant of ST17, which in contrast has been reported in multiple studies throughout the world. Studies report carriage of *bla*_KPC-2_ and *bla*_NDM-1_ in central China [33], blaNDM-7 in Egypt [34] and South Africa 28, and blaOXA-181 in a neonatal ICU in Ghana [35], the same as in the present study. Globally, we found few ST17 isolates closely related to the ST2621 expansion, all originating from TBH. This may indicate a local emergence of a clone acquiring a competitive advantage through acquisition of the carbapenemase. For both locally successful clones, the acquisition of particular plasmids seems to have played a major role in their success. However, with the current data, we cannot determine exact plasmid makeup nor the origin of the plasmids, which is the subject of further investigation. The transmission of ST2621 within the institution occurred over a longer time period compared to ST39. ST2621 was largely confined to the adult wards, which comprise 3-fold more inpatient beds than the neonatal and paediatric wards. The reaction to the outbreak was poor, as limited or no CRE colonization screening and subsequent isolation measures were implemented, which may have contributed to the more protracted course of the outbreak. Although other factors may have played a role in transmission dynamics, the differences in the ST39 and ST2621 outbreaks do (indirectly) support the importance of screening in limiting the spread of these pathogens and guiding the implementation of appropriate IPC measures.

As much as there were parallels between the two lineages – both showing similar diversity and being repeatedly introduced into and transmitted within TBH – they also had interesting differences. Not only did they carry different carbapenemases, but they were also associated with different patient populations. ST39 mainly came from neonatal and paediatric patients and carriage samples. If screening on these wards had not been as extensive, the massive spread of this clone and the associated highly important carbapenemase *bla*_NDM-1_ may have gone undetected for a long time. In contrast, ST2621 was more associated with adult patients and clinical samples. Given that limited CRE colonization screening was undertaken in the adult wards, this almost certainly presents an underestimation of the true spread, and the propensity to cause more disease cannot be quantified sufficiently. Whilst these isolates were obtained from clinical samples, it is possible – and likely – that at least a proportion of them reflect carriage or colonization rather than clinical infection. It is a limitation of this work that retrospective record review was not possible to assess the clinical significance of these isolates.

There were no particular virulence factors present in the isolates that may influence the clinical picture. The K-Locus capsular polysaccharide (CPS) and the O-Locus lipopolysaccharide (LPS) are important determinants of virulence and bacterial interaction with the immune system [36]. Five K loci were identified amongst the four major sequence types. All ST17 and ST2621 isolates were KL25 and ST48 were KL62. While KL2 was dominant in ST39 (147/164, 89.6%), KL62, KL149 and KL23 were also identified. The isolates showed very low virulence scores, with only yersiniabactin detected in the majority of isolates, suggesting that there was no convergence of hypervirulence and carbapenem resistance in the setting. Three yersiniabactin lineages, *ybt*10, *ybt*14 and *ybt*16 were identified, which were embedded in three integrative conjugative elements: ICE*Kp*4, ICE*Kp*4 and ICE*Kp*12, respectively.

The increasing reports of infections caused by *K. pneumoniae* in Africa indicates the need for active surveillance of *K. pneumoniae* clones circulating within hospitals. This is essential to inform IPC practices, treatment guidelines, and antimicrobial stewardship (AMS) programmes. Especially in post-pandemic terms, genomic surveillance is most promising for management of outbreaks, providing the highest resolution for typing and the full information on the bacterial genome including mobile antimicrobial resistance genes for risk assessment [37]. Advances in sequencing technologies and analysis tools have rapidly increased the output and analysis speed as well as reduced the cost of WGS such that several countries are now able to use WGS-based typing as a routine tool for monitoring and detection of MDR pathogens and early detection of outbreaks [38,39]. The biggest barriers to expanded use of WGS in low-middle-income countries are constraints of infrastructure (laboratory and computational), expertise and cost of sequencing. In contrast, PCR-based methods may offer a relatively cheap and rapid tool for bacterial typing and detection of carriage of antimicrobial resistance genes. However, this would need to be informed by WGS, and also supported by WGS at selected times or of selected isolates to ensure the ongoing appropriateness of PCR-based approaches. This will allow monitoring of the strains and their continued dominance. Any relevant changes in the proportion of ST/carbapenemase combination could then also be used for targeted sequencing of these isolates to investigate further changes in the CRKP epidemiology. From an IPC perspective, it would allow for the tracking of further spread of these two dominant clones across the institution and could be used to inform the success of any IPC interventions.

Limitations of the study include differences in the proportion of CRKP clinical and carriage isolates collected over time (isolates from 2018 to 2020 included both clinical and carriage isolates, however, isolates received from the NICD from 2016 to 2017 were clinical only) and due to the COVID-19 pandemic, not all CRE isolates were collected in 2020, particularly carriage isolates which would probably have been mainly from the paediatric and neonatal patient population. The isolate collection was a convenience sample, and we did not have access to all the isolates. There was also no sustained program of swabbing (CRE screening) of every patient which would have been useful in terms of tracking transmission. This lack of consistent swabbing prevented us from assessing how many patients were colonised on admission compared to how many acquired CRE during the course of their hospital stay. Another limitation is that some isolates may have been missed due to non-expression of carbapenemase genes, and phenotypic susceptibility testing may have missed some isolates, as not all carbapenemase production results in phenotypic non-susceptibility. This is something that has been described with OXA-48-like enzymes in particular [40]. Another limitation is that the differences in sample types may be artefact of how the screening was carried out. We also acknowledge that public databases such as Pathogenwatch are limited in the geographic availability, the bias towards sequencing more highly-resistant isolates, and the time at which sequencing data is available. We may also be detecting the described lineages as “local lineages” in Cape Town mainly because of the absence of data from other hospitals and regions that are geographically close. Future research should include surveillance to identify and describe these clones (ST2621 and ST39) in isolates from other institutions, to determine the extent of their dissemination, plasmid characterisation and dissemination in other species.

## Conclusions

None of the common international clones were reported in our study, except for a single ST307 isolate. Instead, two clones, ST2621 and ST39 predominated in the CRKP isolates. Both clones appear to have emerged locally through the acquisition of different carbapenemase carrying plasmids. Both clones had been circulating within the institution since 2016 and possibly earlier. So far, ST2621 is an uncommon clone worldwide, without any published full genome data. It is a single locus variant of ST17 that, similar to ST39, is a globally important lineage. The epidemiological picture at this institution was not of large, single source outbreak but rather of multiple smaller endemic clones with multiple introductions of CRKP. The reason for the surge in ST2621 and ST39 during this period is unclear but may be attributable to several factors such as changes or breakdown in hospital IPC practices or staffing. Strict IPC and AMS measures that are implemented hospital-wide are needed to contain ongoing CRKP transmission.

## Materials and methods

### Ethics statements

This study was approved by the Stellenbosch University’s Health Research Ethics Committee (HREC) (ethics reference: S21/01/005). A waiver of individual informed consent was granted for the study as the retrospective data and isolate collection extended to 2016, and it was not feasible to contact historically affected patients. All patient data was de-identified from the outset and only the investigators have access to the database.

### Study site and CRE isolates

Tygerberg Hospital is a 1384-bed tertiary hospital in the Eastern Metropole of Cape Town. The institution provides tertiary services to approximately 3.5 million people from the Western Cape Province [41]. Carriage isolates were from samples (predominantly rectal swabs) sent to the laboratory as part of active screening during the CRE outbreak. The clinical isolates were obtained from different specimen types including blood cultures, urine, cerebrospinal fluid (CSF), abscess, aspirate, respiratory, catheter, swab and tissue submitted from the adult, paediatric and neonatal wards at the institution. The ward to which a patient was admitted was used to define the age/population classification.

All isolates were from routinely collected samples, and only CRE isolates that had been kept aside by the laboratory were included in this study, as a convenience sample. Non-duplicate carbapenem resistant *K. pneumoniae* isolates representing both carriage (rectal swab or stool) and infection (clinical samples) were collected from patients at the institution from 2016 to 2020 (S1 Table). In instances where patients had multiple isolates from different specimens, only the first clinical isolate and the first carriage isolate from a patient were included, when available. Before the outbreak (August 2018), no carriage isolates were collected, and during the initial SARS-CoV-2 pandemic lockdown in 2020, only clinical CREs were collected as part of the National Institute for Communicable Diseases (NICD) surveillance.

Bacterial identiﬁcation to the species level and routine AST were performed on the clinical isolates as part of routine diagnostic practice by the Tygerberg Hospital NHLS diagnostic laboratory using the Vitek2 (BioMérieux, France) automated system and interpreted based on the Clinical & Laboratory Standards Institute (CLSI) guidelines of the relevant years. Isolates non-susceptible to carbapenems on routine susceptibility testing were defined as CRE. Screening samples were cultured on the selective chromogenic media bi-plate ChromID CARBA SMART (BioMérieux, France) to screen for OXA-48 on one side and KPC and NDM-1 on the other, among carbapenemase-producing Enterobacterales; the same procedures were then followed for species identification and AST.

### Real time PCR for detection of carbapenemase genes

The presence of carbapenemases was determined using a modification of a previously described real-time multiplex PCR assay [42], incorporating *bla*_IMP_ primers [43]. Two different multiplex reactions were used: one to detect *bla*_GES_, *bla*_NDM_ and *bla*_VIM_, the second for *bla*_OXA-48_, *bla*_IMP_ and *bla*_KPC_. All primers were used at a final concentration of 0.2 µM. The 2 × high-resolution melt (HRM) PCR Master Mix (Qiagen, Germany) was used for amplification.

### Whole genome sequencing and quality control

The isolates were sequenced using the Illumina MiSeq sequencing platform at the Medical Center University of Freiburg. Isolate identification was confirmed using MALDI-TOF mass spectrometry (Bruker, USA) and isolates were plated on ChromID CARBA SMART plates to confirm carbapenem resistance. DNA was extracted using the High Pure PCR template preparation kit (Roche diagnostics). The sequencing library was prepared using Nextera DNA flex library preparation kit (Illumina) according to manufacturer’s instructions for paired-end sequencing (300 cycle v2, 2 × 150 reads).

### Further bioinformatics analyses

For phylogenetic reconstructions, single-nucleotide polymorphisms (SNPs) were filtered from the mapping data with GATK (https://gatk.broadinstitute.org/hc/en-us), and only SNPs with at least 4 reads coverage and present in >75% of reads were included. These variant filtered files were converted to a fasta file, where SNP sites and absent sites (N) were replaced and compared to the reference genome. All isolates of a species were then combined to an alignment, and regions resembling mobile genetic elements were removed (https://github.com/andrewjpage/remove_blocks_from_aln). Regions of high SNP density indicative of recombination were identified and removed using Gubbins [44]. SNP sites were extracted and the resulting alignment used to reconstruct a maximum likelihood phylogeny with RAxML v8.2.4 [45], and a pairwise SNP distance matrix was generated using snp-dists 0.6.3 (https://github.com/ tseemann/snp-dists). Metadata for global collections of ST17 and ST39 were downloaded from Pathogenwatch. The focus was on human samples, so environmental were excluded as well as samples that were rather genetically distant from our collection. In highly clonal expansions, one or two representatives were selected, always trying to include samples from all the continents to have a good portrayal of the global context. After download of sequencing reads, same mapping approach was used as above for global and local samples with the following references: ST17-Kp2177 (CP075591, chromosome) [23], and an internal reference of ST39 (KP749, assembly accession CAUIDA01) in the absence of a suitable public reference. Phylogeny was also constructed with the maximum likelihood method (RAxML v8.2.4). To describe the transmission chains, a threshold of 2 SNPs between isolates from different patients was used to define a cluster of patients with transmission of CRE isolates.

ABRicate (www.github.com/tseemann/abricate) was used to detect acquired antimicrobial resistance genes and plasmid replicon typing using the ResFinder database provided by the Center for Genomic Epidemiology (http://www.genomicepidemiology.org), and the PlasmidFinder 2.1 software (https://cge.food.dtu.dk/services/PlasmidFinder/) for the local and global collection. Phylogenetic trees were visualized with Figtree, iTol [46] and the interactive Microreact (https://microreact.org/). A virulence score report was generated with Kleborate, which outputs virulence scores ranging from 0 to 5 [47].

Sequencing statistics can be found in S1 Table. Sequencing data has been deposited in the ENA under project number (PRJEB63361), with individual accession numbers listed in S1 Table. Several microreact projects have been created: Overview of all ST39 at TBH (https://microreact.org/project/5ZJRSb6bshjY5f5fmMhzY8-cresast39), Overview of all ST17 Complex at TBH (https://microreact.org/project/bEtHg2DTSNyBvYEHgxu9uz-cresaonlyst172621230711), Global view of ST39 (https://microreact.org/project/k5xuuDEJurmbW4tbUuMzqw-cresaglobalst39final230523), Global view of ST17 Complex (https://microreact.org/project/gw3jhssF7We1ybi3ppixKy-globalcresast172621230906).

## Supporting information

S1 Fig Phylogenetic tree for ST334. Year of isolation, source (clinical or carriage sample), population (adult, neonate, paediatric), and the carbapenemase genes detected are shown.
(TIFF)

S2 Fig Overall phylogeny of the ST39 CRKP isolates from TBH. Inset: close-up view of the second clade of closely related isolates. Collection date, population, source, and carbapenemases are annotated on the phylogeny, as well as the endemic expansion, which is further explored in Fig 3A.
(TIFF)

S3 Fig Plasmid analysis of local isolates of ST39 in a global context. The TBH isolates are highlighted in yellow. Carbapenemase genes and the presence of three plasmids is shown: ColRNAI, IncFIB, and IncFII.
(TIFF)

S4 Fig Overall phylogeny of the ST17 Complex CRKP isolates from TBH. Collection date, population, source, and carbapenemases are annotated on the phylogeny. The endemic expansion is signified by ST2621, which is further explored in Fig 3B.
(TIFF)

S5 Fig Plasmid analysis of local isolates of the ST17 complex in a global context. The TBH isolates are highlighted in yellow. Carbapenemase genes and the presence of the following plasmids is shown: ColRNAI, IncFIA, IncFIB, IncFII, IncR, and IncX3.
(TIFF)

S1 Table Metadata of carbapenem resistant *K. pneumoniae* from South Africa, antibiotic resistance genes summary and virulence summary. (XLSX)
