# Supplementary figures and images for: Carbapenem resistant Klebsiella pneumoniae isolates at a tertiary hospital in Cape Town, South Africa, are dominated by specific local clones rather than previously described international lineages
Source: PLoS Pathog. 2026 Jan 16;22(1):e1013859. doi: 10.1371/journal.ppat.1013859 (PMC12810914; doi:10.1371/journal.ppat.1013859)

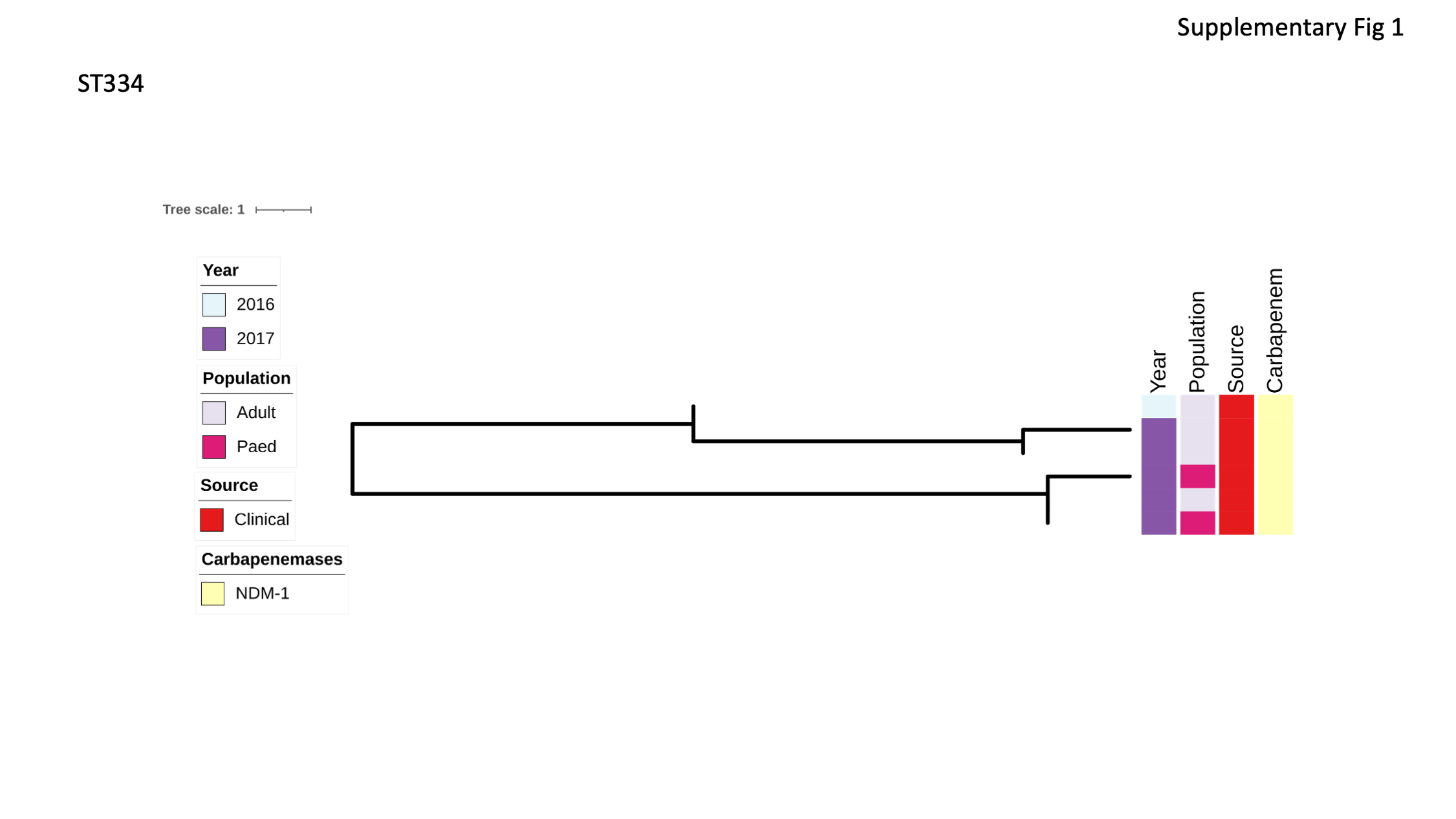

Supplement: S1 Fig — Year of isolation, source (clinical or carriage sample), population (adult, neonate, paediatric), and the carbapenemase genes detected are shown. (TIFF) [file ppat.1013859.s001.tiff]

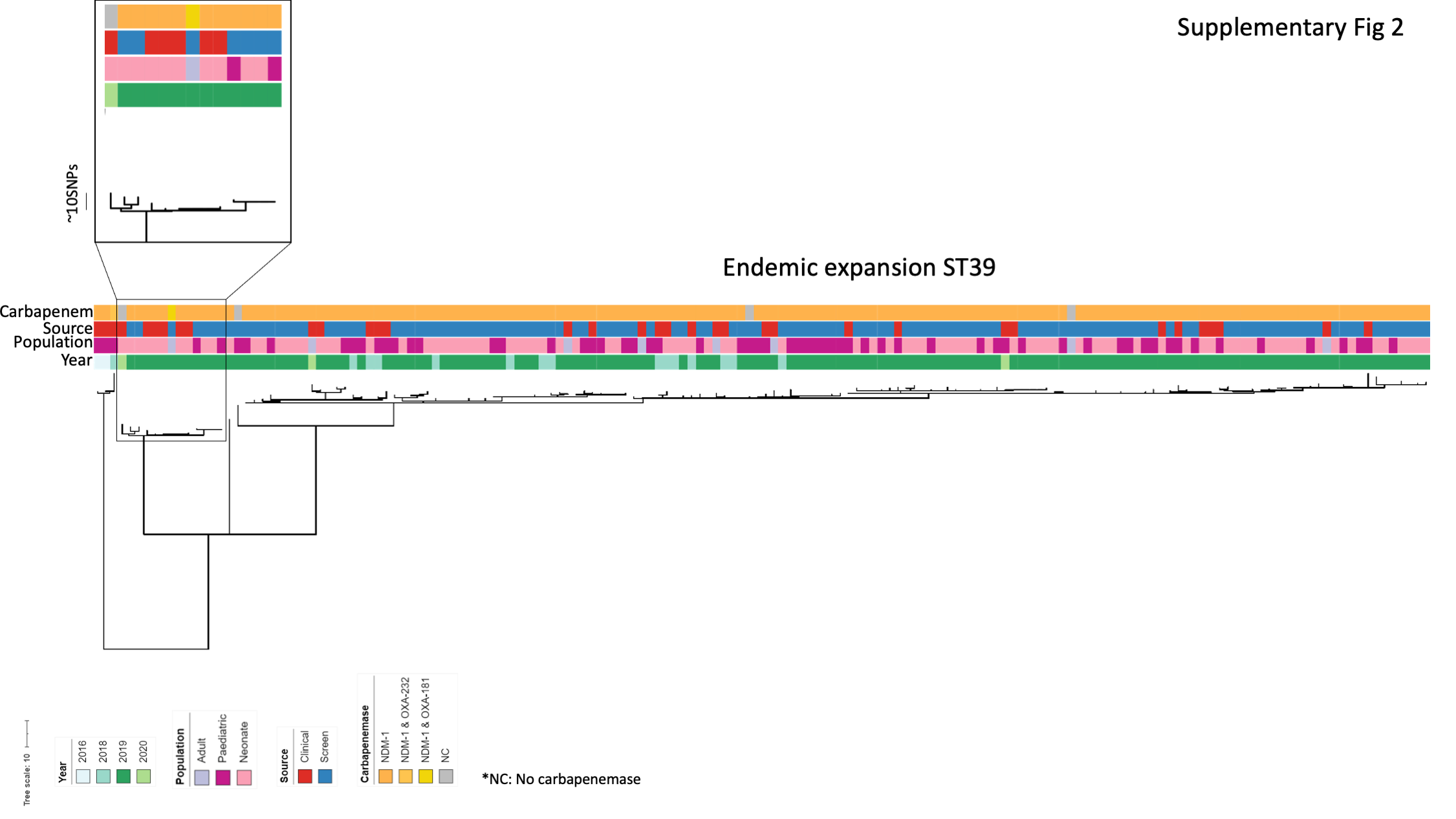

Supplement: S2 Fig — Inset: close-up view of the second clade of closely related isolates. Collection date, population, source, and carbapenemases are annotated on the phylogeny, as well as the endemic expansion, which is further explored in Fig 3A. (TIFF) [file ppat.1013859.s002.tiff]

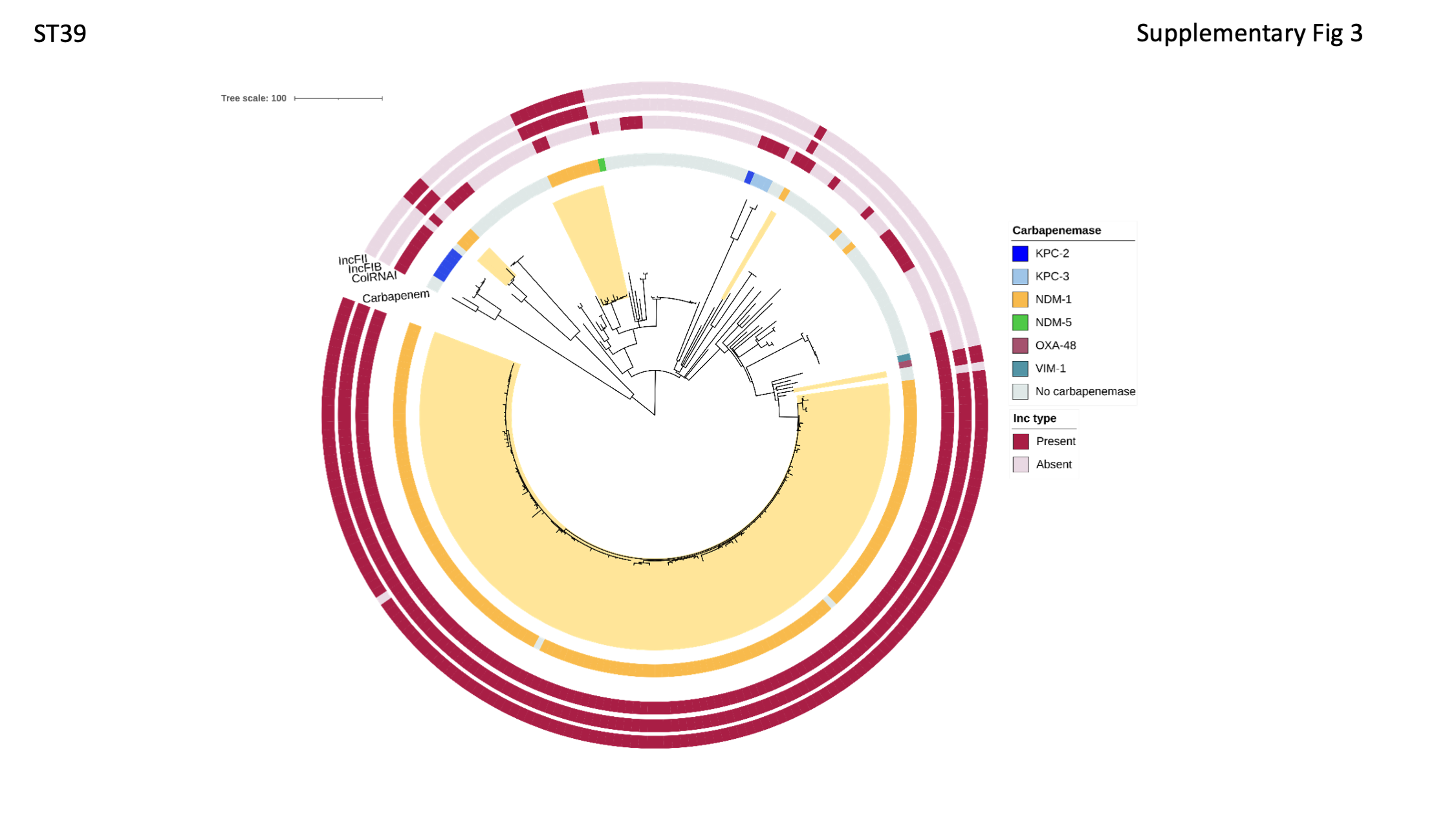

Supplement: S3 Fig — The TBH isolates are highlighted in yellow. Carbapenemase genes and the presence of three plasmids is shown: ColRNAI, IncFIB, and IncFII. (TIFF) [file ppat.1013859.s003.tiff]

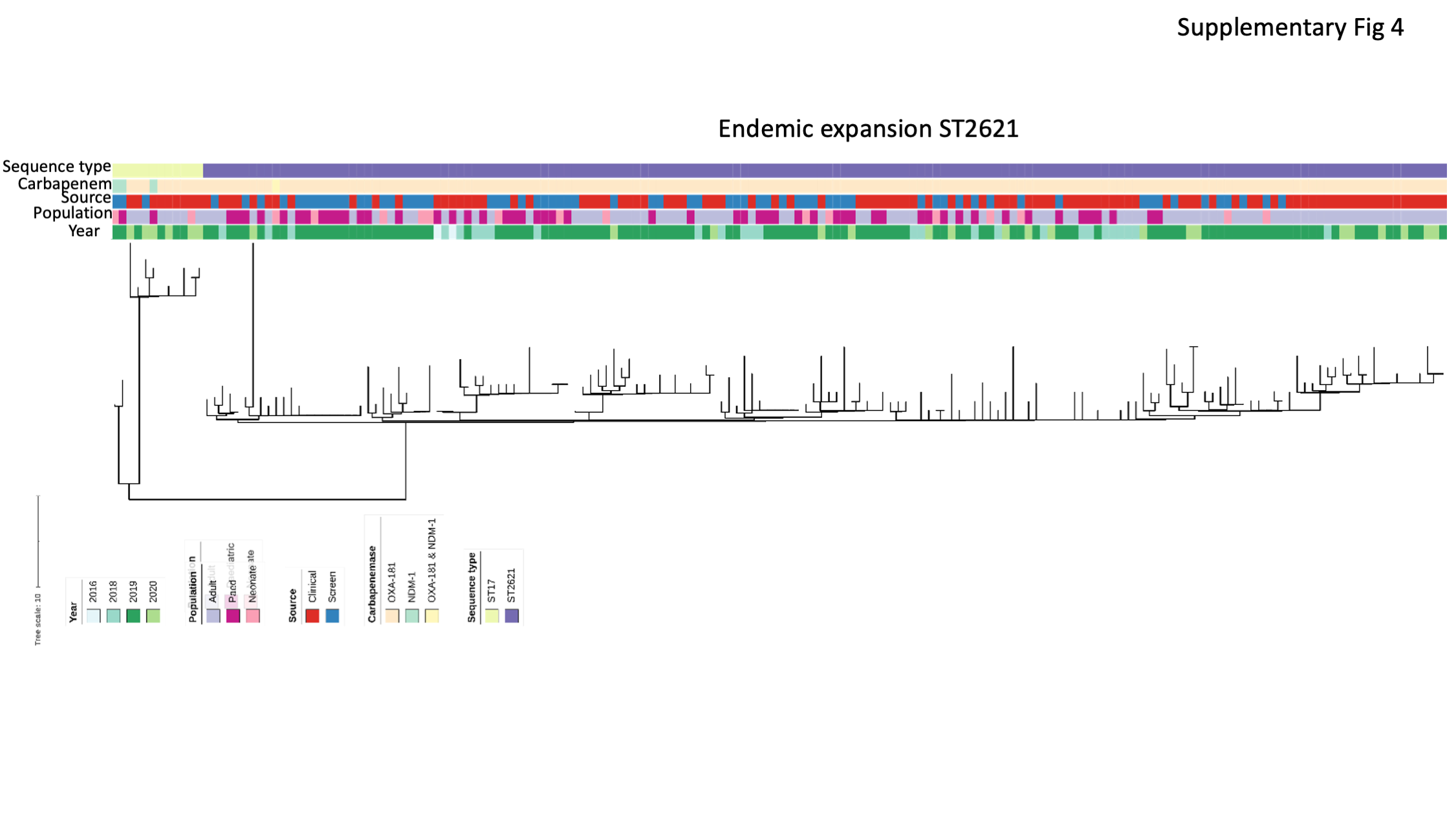

Supplement: S4 Fig — Collection date, population, source, and carbapenemases are annotated on the phylogeny. The endemic expansion is signified by ST2621, which is further explored in Fig 3B. (TIFF) [file ppat.1013859.s004.tiff]

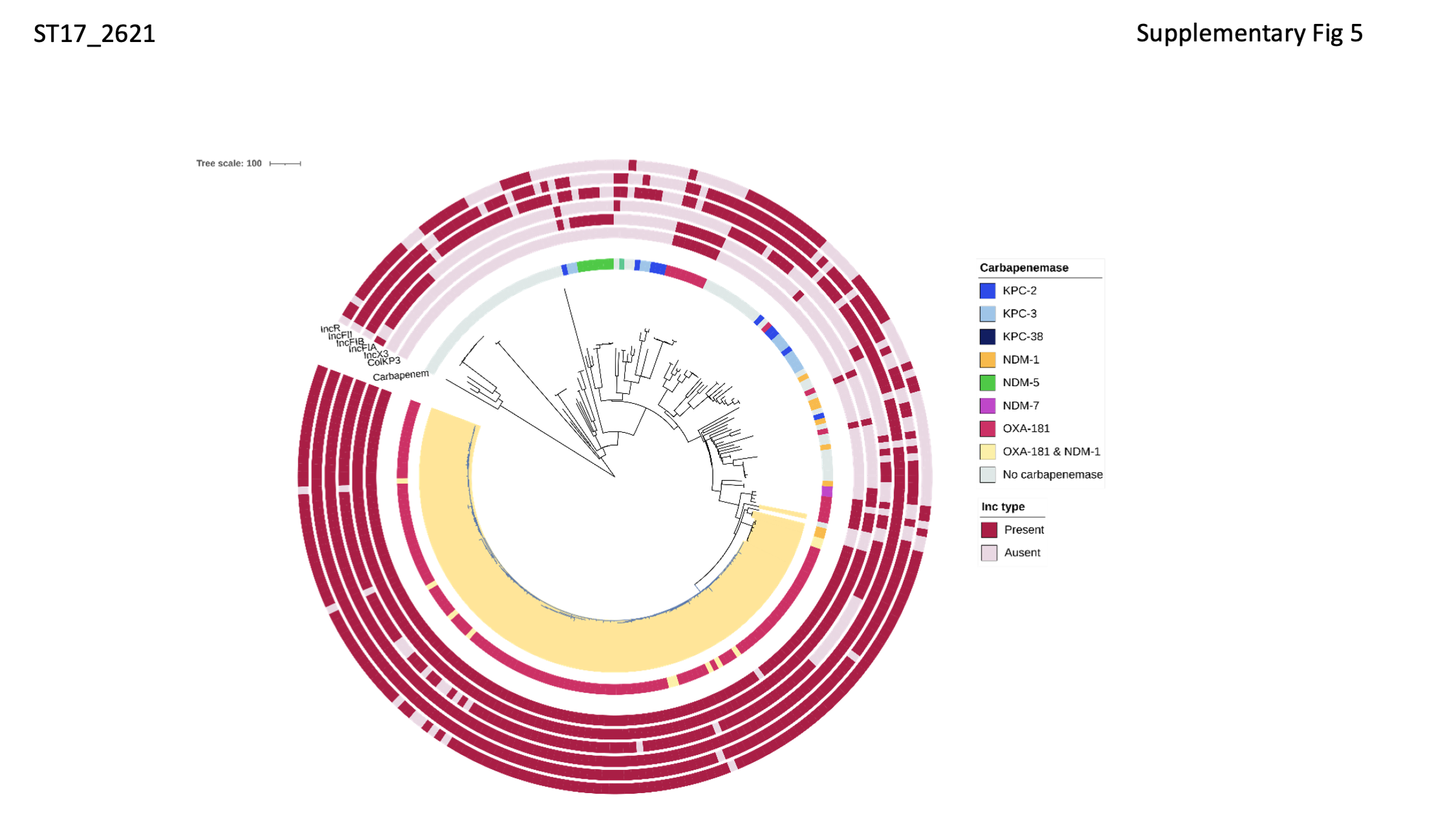

Supplement: S5 Fig — The TBH isolates are highlighted in yellow. Carbapenemase genes and the presence of the following plasmids is shown: ColRNAI, IncFIA, IncFIB, IncFII, IncR, and IncX3. (TIFF) [file ppat.1013859.s005.tiff]
